# The Cholinergic Anti-Inflammatory Pathway Attenuates the Development of Atherosclerosis in *Apoe^-/-^* Mice through Modulating Macrophage Functions

**DOI:** 10.3390/biomedicines9091150

**Published:** 2021-09-03

**Authors:** Zhengjiang Qian, Haiyang Yang, Hongchao Li, Chunhua Liu, Liang Yang, Zehui Qu, Xiang Li

**Affiliations:** 1 Guangdong Provincial Key Laboratory of Brain Connectome and Behavior, CAS Center for Excellence in Brain Science and Intelligence Technology, Brain Cognition and Brain Disease Institute (BCBDI), Shenzhen-Hong Kong Institute of Brain Science-Shenzhen Fundamental Research Institutions, Shenzhen Institute of Advanced Technology, Chinese Academy of Sciences, Shenzhen 518055, China; hy.yang1@siat.ac.cn (H.Y.); hc.li1@siat.ac.cn (H.L.); ch.liu@siat.ac.cn (C.L.); liang.yang@siat.ac.cn (L.Y.); zh.qu@siat.ac.cn (Z.Q.); 2 University of Chinese Academy of Sciences, Beijing 100049, China

**Keywords:** cholinergic anti-inflammatory pathway, atherosclerosis, inflammation, macrophage polarization, foam cell formation

## Abstract

(1) Background: The cholinergic anti-inflammatory pathway (CAP) has been implicated in the regulation of various diseases, including chronic inflammatory cardiovascular disorders such as atherosclerosis (AS). This study aims to explore the underlying regulatory mechanisms of CAP activity in the progression of AS. (2) Methods: The *Apoe^-/-^* mice were subjected to sham, bilateral cervical vagotomy surgery (VGX), and VGX supplemented with Gainesville Tokushima scientists (GTS)-21 (4 mg/kg/d) and then fed with a high-fat diet for 10 weeks. Atherosclerotic lesion size and inflammation levels were investigated by histology and inflammatory cytokines analysis. The blood M1/M2 macrophages were analyzed by flow cytometry. Primary mouse bone marrow-derived macrophages (BMDM), peritoneal macrophages, and RAW264.7 cells were treated with CAP agonists acetylcholine (Ach) and GTS-21 to study their effects on macrophage functions. (3) Results: Compared with the sham group, inhibition of CAP by the VGX resulted in growing aortic lipid plaque area, deteriorated inflammatory levels, and aberrant quantity of M1/M2 macrophages in *Apoe^-/-^* mice. However, these detrimental effects of VGX were significantly ameliorated by the reactivation of CAP through GTS-21 treatment. The in vitro study using macrophages revealed that stimulation with CAP agonists suppressed M1, but promoted M2 macrophage polarization through the upregulation of TNFAIP3 and phosphorylation STAT3 levels, respectively. Moreover, the activation of CAP inhibited the formation of macrophage foam cells in the peritoneal cavity by regulating genes related to cholesterol metabolism. (4) Conclusions: This study provides novel evidence and mechanisms that the CAP plays an important role in the regulation of AS development by controlling macrophage functions, implying a potential use of CAP activation as a therapeutic strategy for AS treatment.

## 1. Introduction

Over the past two decades, the cholinergic anti-inflammatory pathway (CAP) has emerged as an important neuroimmunoregulatory pathway that bridges the immune system and the nervous system [1,2,3,4,5]. Via an inflammatory reflex of the CAP, the vagus nerve perceives the peripheral inflammatory signal and then releases the neurotransmitter acetylcholine (Ach), which stimulates alpha 7 nicotinic acetylcholine receptor (α7nAchR) in multiple immune cells such as macrophage, and eventually inhibits the production of pro-inflammatory cytokines and cytokine-related devastating effects [6,7,8]. It has been widely studied that the activation of CAP, through stimulating the vagus nerve or activating α7nAchR receptor by a specific cholinergic agonist can effectively attenuate the development of various inflammatory diseases, including endotoxemia, rheumatoid arthritis, ischemia/reperfusion injury, and hemorrhagic shock [9,10,11,12,13,14]. Recent studies indicate that CAP may also play a functional role in the regulation of cardiovascular diseases such as atherosclerosis (AS), a chronic inflammatory condition [15,16,17,18]. For instance, maintenance of Ach content by suppressing the activity of acetylcholinesterase with donepezil essentially inhibits atherogenesis in *Apoe^-/-^* mice [19]. Similarly, the application of selective CAP agonists is effective in reducing pro-inflammatory cytokines and improving the pathological features and survival of AS mice [15,20]. By contrast, blocking CAP by the deletion of α7nAchR in bone marrow showed controversial results that either decrease or increase inflammatory status and aortic lesions in different phases of AS development [21,22,23,24]. Although amounts of progress have been achieved, the underlying molecular mechanisms by which CAP regulates inflammatory responses and AS progression are far from being elucidated.

Macrophages, as the predominant immune cell type in atherosclerotic lesions, play a pivotal role in all stages of AS, from lesion initiation to plaque rupture [25,26,27,28,29,30]. Based on the different activation, macrophages are mainly classified into two phenotypes, namely the classically activated M1 and alternatively activated M2 macrophages [31,32]. The M1 macrophages, which are stimulated by Toll-like receptor ligands (e.g., LPS and interferon-γ), have host defense function and produce proinflammatory cytokines and chemokines such as TNF-α, IL-1β, and IL-6 [26]. By contrast, the anti-inflammatory M2 macrophages as induced by Th2-type cytokines (e.g., IL-4 and IL-13) are associated with tissue repair, wound healing, and endocytic clearance by secreting reparative cytokines and enzymes, including IL-10, arginase 1 (Arg1), and transforming growth factor (TGF)β [33]. A number of studies demonstrated that M1 macrophages potentiate, while M2 macrophages ameliorate the development of AS [34,35]. Moreover, it is presumed that the M1 and M2 phenotypes of macrophages can be plastically transformed in response to different microenvironmental conditions and stimulation [36,37].

Besides, macrophages act as the main source for foam cells, facilitating the balance of lipid metabolism in the development of AS [38,39,40,41,42]. In macrophage-derived foam cells, the scavenging of oxidized low-density lipoprotein (ox-LDL) and cholesterol are controlled by a series of genes [38,41,42,43]. For instance, the uptake of ox-LDL is mainly regulated by scavenger receptors such as CD36 and SR-A [41,43]. Acyl coenzyme A: cholesterol acyltransferase-1 (ACAT1) and neutral cholesteryl ester hydrolase (nCEH) are involved in the formation of cholesterol esters. The efflux of cholesterol from macrophages was largely mediated by ATP-binding cassette (ABC) transporters ABCA1 and ABCG1 [41]. It has been shown that aberrant expression of these genes could essentially contribute to the formation of foam cell and lipid accumulation [44,45]. Therefore, the discovery of mechanisms that regulate the functions of macrophages and foam cell formation would be a promising therapeutic target for the treatment of AS [46,47,48,49].

In the present study, we showed that inhibition of CAP by bilateral cervical vagotomy surgery (VGX) facilitated the development of inflammation and AS pathologic features in *Apoe^-/-^* mice, while reactivation of CAP by the treatment of GTS-21 remarkably partially rescued the adverse effects of VGX. An in vitro study showed that the application of CAP agonists, Ach and GTS-21, inhibited M1 macrophage polarization by the upregulation of TNFAIP3, and promoted M2 macrophage polarization through activation of p-STAT3 in primary BMDM and RAW264.7 cells. Moreover, the formation of macrophage foam cells in the peritoneal cavity was also inhibited by the activation of CAP. Overall, our results demonstrated that the CAP plays a crucial role in AS progression by regulating macrophage functions, providing a potential strategy for CAP in the treatment of AS.

## 2. Materials and Methods

### 2.1. Animals and Treatments

All experimental procedures were performed according to protocols approved by the Institutional Animal Care and Use Committee (SIAT-IACUC-20190715-NS-NTPZX -QZJ-A0603) at Shenzhen Institute of Advanced Technology (SIAT), Chinese Academy of Science (CAS). The *Apoe^-/-^* mice with the background of C57BL/6JNj were purchased from Nanjing Biomedical Research Institute of Nanjing University (Nanjing, China) and bred in specified-pathogen-free facilities at SIAT. At the age of 6–8 weeks old, male *Apoe^-/-^* mice were randomly divided into three experimental groups, i.e., sham, bilateral cervical vagotomy surgery (VGX), and VGX supplemented with Gainesville Tokushima scientists (GTS)-21. The VGX was performed with modification as described previously [1,50]. Briefly, mice were deeply anesthetized with 1% pentobarbital sodium (60 mg/kg, body weight) and then were placed in a stereotaxic device. The skin of the neck was shaved, and the surgical site was swabbed with 10% povidone-iodine, followed by 70% ethanol. Left cervical vagotomy was firstly performed by a small skin incision on the neck and the vagus nerve—which is next to the carotid artery—was exposed and resected under a light microscopic identification. The incision was then closed with skin sutures. After one week of recovery, the right cervical vagotomy was conducted with the same surgery procedure above. The sham operation group was carried out in the same way except that the vagus nerve was left intact. After surgery, the mice were fed with a high-fat diet (20% fat, 1.25% cholesterol) for 10 weeks. Treatment of GTS-21 (4 mg/kg) was conducted by intraperitoneal injection bi-daily every 3 days [20].

### 2.2. Serum Lipid and Cytokines Analysis

At the end of treatment, mice were weighed and sacrificed for serum lipid content and inflammatory cytokines analysis. Total cholesterol (TC) and triglyceride (TG) levels in serum were determined using a Micro Content Assay Kit obtained from Solarbio Life Science and Technology Co. (Beijing, China). The OD for TC and TG in the microplate was read at 500 nm and 420 nm, respectively. The concentrations of serum TNFα, IL-1β, and IL-6 were determined using commercially available ELISA kits for mice (4Abio., Beijing, China). The OD of the microplate was read at 450 nm. All the experimental procedures were performed according to the manufacturers’ instructions.

### 2.3. Oil-Red-O Analysis of Atherosclerotic Lesion

The heart along with the aorta was carefully dissected after the mice were perfused with PBS followed by 4% paraformaldehyde (PFA). Lipid content in the thoracic aorta was measured by en face analysis. All fat and connective tissue were removed from the outer layers of the aortas vessel. Then, the aorta was cut longitudinally, stained with Oil-Red-O (ORO, Cat#1320-06-5, Sigma-Aldrich, Saint Louis, MO, USA) followed by washing and mounting on a silicone-coated dish [15,20]. Aortas images were captured using a high-resolution camera. For analysis of atherosclerotic plaque lesions, hearts with aortic root were embedded in Tissue-Tek O.C.T. compound (Cat#4583, Sakura Finetek, Tokyo, Japan) and processed for cryo-sections at 20 μm transverse serial sections. Sections showing the three aortic valves were collected for lipid depositions analysis by ORO staining on Section 3, Section 4 and Section 5 per mouse [20]. After washing, the tissues were mounted on glass slides and imaged by Olympus VS120 virtual microscopy slide scanning system with a high-power-filed image (20×). Lesion lipid quantification of ORO staining was performed using Image J software.

### 2.4. Flow Cytometry

Blood samples from each group of experimental mice were collected via cardiac puncture into EDTA-lined tubes at the end of treatment. For flow cytometry analysis, red blood cells were lysed using Red Blood Cell Lysis Buffer (Cat# 00-4333-57, Thermo Fisher Scientific, Waltham, MA, USA), and then the white cells were pelleted by centrifugation at 400× *g* for 5 min at 4 °C. The obtained cells were sufficiently washed and then stained successively with anti-CD45 FITC (Cat#553080 1:100), anti-CD11b (Cat#557657, 1:100), and anti-CD206 (Cat#565250, 1:100) on ice for 30 min. The samples were performed on a BD FACSCanto II Flow Cytometer. All the antibodies were purchased from BD Pharmingen Bioscience. The data were analyzed with Flowjo software (Version 10.0, Treestar, OR, USA), and M1/M2 macrophages were identified as CD45^+^CD11B^+^CD206^−^/CD45^+^CD11B^+^CD206^−^, respectively.

### 2.5. Cell Culture and Treatment

The mouse RAW264.7 macrophage cells were obtained from the American Type Culture Collection (ATCC, Manassas, VA, USA) and routinely cultured in Dulbecco’s modified Eagle’s medium supplemented 10% heat-inactivated FBS, 1% penicillin/streptomycin at 37 °C in a humidified atmosphere with 5% CO_2_. The murine bone marrow-derived macrophages (BMDMs) were isolated and cultured as described previously [51]. In brief, *Apoe^-/-^* mice at age of 6–8 weeks were sacrificed and the tibiae and femora were isolated under sterile conditions. The marrow was repeatedly flushed from the bones and then filtered through a 40-µm nylon cell strainer followed by centrifugation at 1000× *g* for 5 min. The resulting cell pellet was resuspended and differentiated into macrophages in RPMI medium supplemented with 10% FBS, 1% penicillin/streptomycin in the presence of 10 ng/mL M-CSF (Cat#NBP2-35165, Novus, Novus, CO, USA) for 7 days.

Macrophage polarization was performed as described previously [32,51]. For M1 activation, cells were stimulated with lipopolysaccharide (LPS, 500 ng/mL; Sigma-Aldrich, Saint Louis, MO, USA) or IFNγ (50 ng/mL; R&D Systems, Minneapolis, Minnesota, USA), and for M2 activation, cells were stimulated with IL-4 (20 ng/mL; PeproTech, Cranbury, NJ, USA). The polarized M1 or M2 phenotype macrophages were further incubated with Ach or GTS-21 at the indicated concentration for 24 h. To inhibit acetylcholinesterase activity, pyridostigmine bromide (1 mM) was administrated in an Ach treatment experiment [1]. The effects of Ach and GTS-21 on M1/M2 macrophage function were determined by the analysis of M1 and M2 maker genes (i.e., TNFα, IL-1β, IL6 for M1 phenotype, and Arg1, TGFβ, IL10, mannose receptor (Mrc1), resistin-like α (Retnla) for M2 phenotype, respectively).

### 2.6. Evaluation of Foam Cell Formation

Both in vivo and in vitro foam cell formation were evaluated as described previously [45]. For in vivo evaluation, the three experimental groups of *Apoe^-/-^* mice (sham, VGX, and VGX + GTS-21) were injected intraperitoneally with 0.5 mL of 4% thioglycollate, and peritoneal macrophage cells were collected after 3 days. By adhering for 2–3 h, the adherent macrophages were either stained with ORO or used to measure total cholesterol. For in vitro evaluation of foam cell formation, peritoneal macrophages were isolated, serum-starved for 12 h, and then incubated with 50 μg/mL OxLDL (Cat# L34357, Thermo Fisher Scientific, Waltham, MA, USA) for 48 h in the presence or absence of CAP agonists. After that, cells were collected for ORO staining or gene expression analysis. The procedure for cell ORO staining was performed as described previously [52]. In brief, macrophages were first fixed with 4% PFA for 10 min, stained with ORO for 15 min, followed by rinsing with 60% isopropanol and PBS 3 times. The positive-staining cells were observed via a light Zeiss microscope system (Axio observer 3) with at least 10 fields (20×) in each sample.

### 2.7. RNA Extraction and qRT-PCR

Total RNA from cells or tissue was extracted using TRIZOL Reagent (Invitrogen, Carlsbad, CA, USA) according to the manufacturer’s instructions. The concentration of RNA was quantified by the NanoDrop 2000c Spectrophotometer (Thermo Fisher Scientific, Waltham, MA, USA). The first strand of cDNA was synthesized from 1 μg of total RNA using One-Step gDNA Removal and cDNA Synthesis SuperMix (TransGen Biotech, Beijing, China). The quantitative PCR experiments were conducted on QuantStudio Real-Time PCR system (Applied Biosystems, Foster City, CA, USA) using TransStart^®^ Tip Green qPCR SuperMix (TransGen Biotech, Beijing, China) with gene-specific primers. The GAPDH was used as an internal control for normalization. All reactions were performed in triplicate with three independent experiments, and the relative expressions of mRNA levels were calculated using the 2^−△△Ct^ method. The stability of GAPDH expression in different samples is compatible with MIQE guidelines as previously described [53]. Primers sequences used in this study were presented in Table 1.

### 2.8. Western Blotting

Protein extraction and a western blotting assay were conducted as described previously [54,55]. Total protein was extracted with RIPA lysis buffer supplemented with protease inhibitor cocktail (Roche, Basel, Switzerland). The protein level of each sample was measured using a BCA protein assay kit (Thermo Fisher Scientific, Waltham, MA, USA). Equal protein amounts (30 μg) were loaded for sodium dodecyl sulphate-polyacrylamide gel electrophoresis (SDS-PAGE) and transferred to a polyvinylidene difluoride (PVDF) or nitrocellulose membrane. After blocking the nonspecific site with 5% non-fatted milk for 1 h, the membrane was incubated with specific primary antibody overnight at 4 °C, including anti-NF-κB-p65 (Cat#ab16502, Abcam, Cambridge, UK), anti-NF-κB-p-p65 (S536) (Cat# ab76302, Abcam, Cambridge, UK), anti-STAT6 (Cat#A0755, Abclonal, Boston, MA, USA), anti-pSTAT6 (Y641) (Cat#AP0456, Abclonal, Boston, Massachusetts, USA), anti-STAT3 (Cat#4904, CST, Boston, MA, USA), anti-pSTAT3 (Y705) (Cat#9145, CST), anti-Arg1 (Cat#66129-1-Ig, Proteintech, Wuhan, China), anti-TNFAIP3 (Cat#23456-1-AP, Proteintech, Wuhan, China), anti-GAPDH (Cat# 10494-1-AP, Proteintech, Wuhan, China), and then incubated with horseradish peroxidase-conjugated secondary antibody for 1 h at room temperature. The immune-blotting signals were visualized with an ECL kit (West-Pico, Super Signal; Pierce, Rockford, IL, USA) using the Tanon-5200Multi chemiluminescent imaging system (Shanghai, China).

### 2.9. Transfection of Small Interfering RNA (siRNA)

SiRNAs of the target genes, including siTNFAIP3 (Cat# sc-37656), siSTAT3 (Cat# sc-29494), siCD36 (Cat# sc-37245), siABCG1 (Cat# sc-41139), and the corresponding negative control RNAs (si-NC) were purchased from Santa Cruz Biotechnologies. Transient transfection of these siRNAs into BMDM cells was performed using Lipofectamine™ RNAiMAX Transfection Reagent (Cat# 13778075, Invitrogen, Carlsbad, CA, USA) according to manufacturer’s instructions. Macrophages were transfected with 20 nM siRNA and the cells were used for analysis after 24–48 h of transfection.

### 2.10. Cholesterol Efflux Assay

The cholesterol efflux assay was performed as previously described with modification [56]. Briefly, the isolated peritoneal macrophage cells were serum-starved and loaded with 10 μg/mL NBD-cholesterol (22–(N-(7-nitrobenz-2-oxa-1,3-diazol-4-yl) amino)–23,24-bisnor-5-cholen-3β-ol, Cat# N1148, Thermo Fisher) for 24 h. Then, the culture medium was removed and cells were washed and incubated in RPMI 1640 medium for an additional 24 h in the presence or absence of GTS-21. The NBD cholesterol level released from the cells was determined using a microplate reader (excitation at 469 nm, emission at 537 nm). The cholesterol efflux ratio was calculated as follows: cholesterol efflux ratio = (NBD cholesterol in medium/(NBD cholesterol in medium + NBD cholesterol in cells)) _GTS-21_/(NBD cholesterol in medium/(NBD cholesterol in medium + NBD cholesterol in cells))_control_.

### 2.11. Statistical Analysis

Statistical analyses were performed with the SPSS version 13.0 software 261 packages (SPSS, Chicago, IL, USA) for Windows. All data are presented as mean ± SD unless otherwise stated. When only two groups were compared, the statistical differences were assessed with the double-sided Student’s *t*-test. The number of samples per group is stated in the figure legends. Comparisons among multiple groups were performed using one-way ANOVA with Tukey’s post hoc test. Two-way ANOVA was used for the analysis of multiple groups with Tukey’s multiple comparison post hoc test. For all experiments, *p*-value < 0.05 was considered a significant difference.

## 3. Results

### 3.1. Effect of CAP Activity on Lipid Plaque Formation and Inflammatory Responses

**Figure 1:**
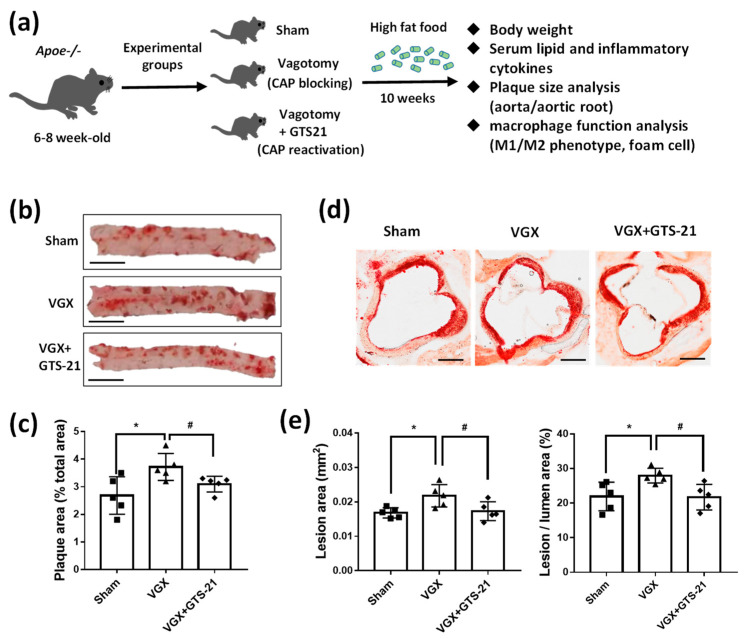

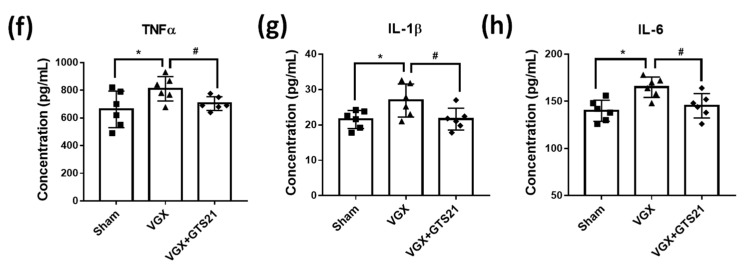
Effects of CAP activity on the development of atherosclerotic plaque and inflammation level in *Apoe^-/-^* mice. (**a**) The *Apoe^-/-^* mice were subjected to three experimental groups, i.e., sham, bilateral cervical vagotomy surgery (VGX) alone, and VGX supplemented with GTS-21 (VGX + GTS-21). The three groups of mice were fed with a high-fat diet for 10 weeks and then used for analysis. (**b**) Oil-red-O (ORO) staining of *en face* preparations of the thoracic aorta. Representative samples are shown. Scale bars represent 200 mm. (**c**) Quantitative analysis of the atherosclerotic plaque area in the aorta. Data are presented as means ± SD (*n* = 5 mice). * *p* < 0.05 as compared to sham; ^#^ *p* < 0.05 as compared to VGX; ns indicates not significant. (**d**) The aortic arch was sectioned and stained with ORO. Representative samples are shown. Scale bar represents 100 μm. (**e**) Quantitative analysis of the atherosclerotic lipid lesion in the aortic root. (Average of 3–5 sections per mouse). Data are presented as mean ± SD (*n* = 18–20 sections). * *p* < 0.05 as compared to sham; ^#^ *p* < 0.05 as compared to VGX; ns indicates not significant. (**f**–**h**) Serum levels of TNFα (**f**), IL-1β (g), and IL-6 (**h**) in each group of experimental animals. Data are presented as mean ± SD (*n* = 6 mice). * *p* < 0.05 as compared to sham; ^#^ *p* < 0.05 as compared to VGX; ns indicates not significant.

To investigate the role of CAP in the development of AS, adult male *Apoe^-/-^* mice were randomly divided into three experimental groups, (i) sham group, i.e., on mice, a comparable surgical procedure was performed in which the vagus nerves were isolated but not transected; (ii) CAP blocking group, i.e., mice were subjected to bilateral cervical vagotomy (VGX); and (iii) reactivation of CAP group, i.e., mice were subjected to VGX following by the injection of GTS-21, an agonist of CAP (Figure 1a). After 10 weeks of a high-fat diet, the *Apoe^-/-^* mice showed robust pathological lipid lesions in the aorta compared to wild-type mice (Supplementary Figure S1), confirming the essential atherosclerotic features in *Apoe^-/-^* mice. In the three experimental groups, mice with VGX presented significantly enhanced lipid deposition in the thoracic aorta (Figure 1b,c) and expanded atherosclerotic lesion plaques in the aortic arch (Figure 1d,e) as compared with the sham group. However, these lipid accumulations in the aorta as induced by VGX were significantly inhibited by the GTS-21 supplementation (Figure 1b–e). Moreover, the analysis of serum cytokines revealed that pro-inflammatory factors such as TNFα, IL-1β, and IL-6 were significantly increased in VGX mice compared with the sham group, while the VGX caused a high release of pro-inflammatory factors were significantly suppressed by GTS-21 treatment (Figure 1f–h). Together, these data suggest that the activity of CAP regulates atherosclerotic plaque formation and inflammation level in *Apoe^-/-^* mice.

### 3.2. Effect of CAP Activity on the Abundance of M1 and M2 Macrophages

**Figure 2:**
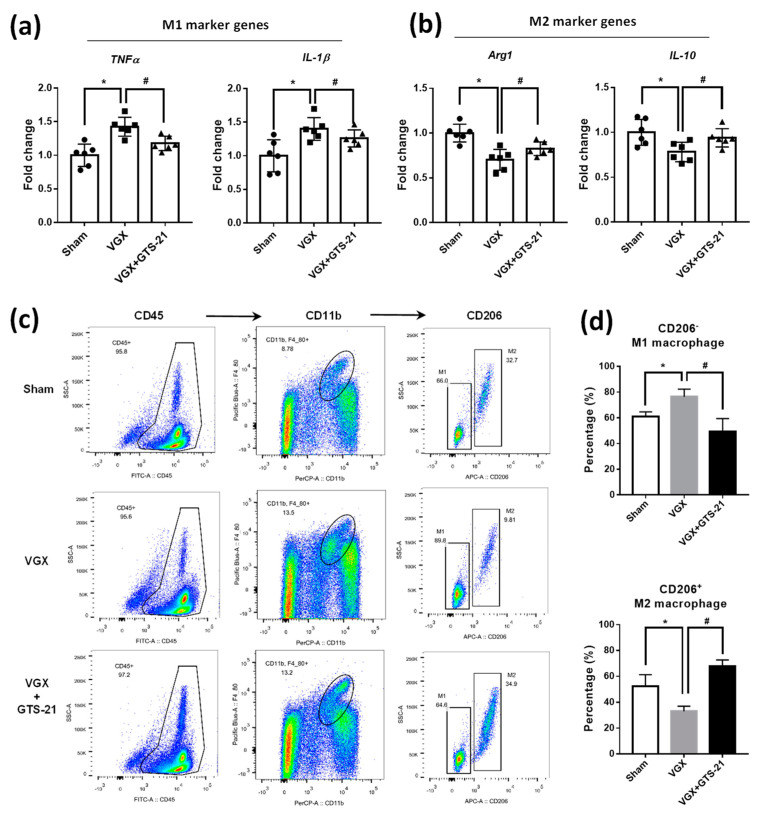
Effects of CAP activity on the quantity of M1 and M2 macrophages. (**a**,**b**) The mRNA expression analysis of M1 pro-inflammatory genes, i.e., *TNFα* and *IL-1β* (**a**), and M2 pro-reparative genes, i.e., *Arg1* and *IL-10* (**b**), in plaques of the thoracic aorta. Data are presented as mean ± SD (*n* = 6 mice), * *p* < 0.05 as compared to sham; ^#^ *p* < 0.05 as compared to VGX; ns indicates not significant. (**c**) Representative images showing flow cytometry analysis of M1 and M2 macrophage in the blood of the three experimental mice. The CD45^+^CD11b^+^CD206^−^ cells were classified as M1 macrophage, while the CD45^+^CD11b^+^CD206^+^ cells were identified as M2 macrophage. (**d**) Relative quantification of the change in the number of M1 (up panel) and M2 (down panel) macrophages. Data are presented as mean ± SD (*n* = 3 mice). * *p* < 0.05 as compared to sham; ^#^ *p* < 0.05 as compared to VGX; ns indicates not significant.

Since macrophage polarization is crucial for the inflammation level in the pathogenesis of AS [25,26], we asked whether the activity of CAP influences the M1 and M2 subtypes of macrophage. We first determined the macrophage phenotype in plaques of the thoracic aorta by analyzing their specific markers. The results showed that VGX mice had higher expression of M1 marker genes (i.e., *TNFα* and *IL-1β*) but lower M2 marker genes (i.e., *Arg-1* and *IL-10*) (Figure 2a,b); while the expression of these genes was partially reversed in VGX mice supplemented with GTS-21 (Figure 2a,b). In addition, the M1 and M2 macrophages were further quantified in the blood. The white cells were successively labeled with antibodies including anti-CD45, anti-CD11b, and anti-CD206 to finally obtain M1 and M2 macrophage subsets (Figure 2c). We found that the VGX mice showed an increased number of M1 macrophages and decreased M2 macrophages in circulation, whereas inverse changes of M1 and M2 macrophages were observed in VGX mice treated with GTS-21 (Figure 2d). Thus, these findings suggest that the CAP activity may alter the abundance and residence of M1 and M2 macrophages, which potentially contribute to the inflammatory responses and lipid plaques in AS development.

### 3.3. Effects of CAP Agonists on M1 Macrophage Polarization

**Figure 3:**
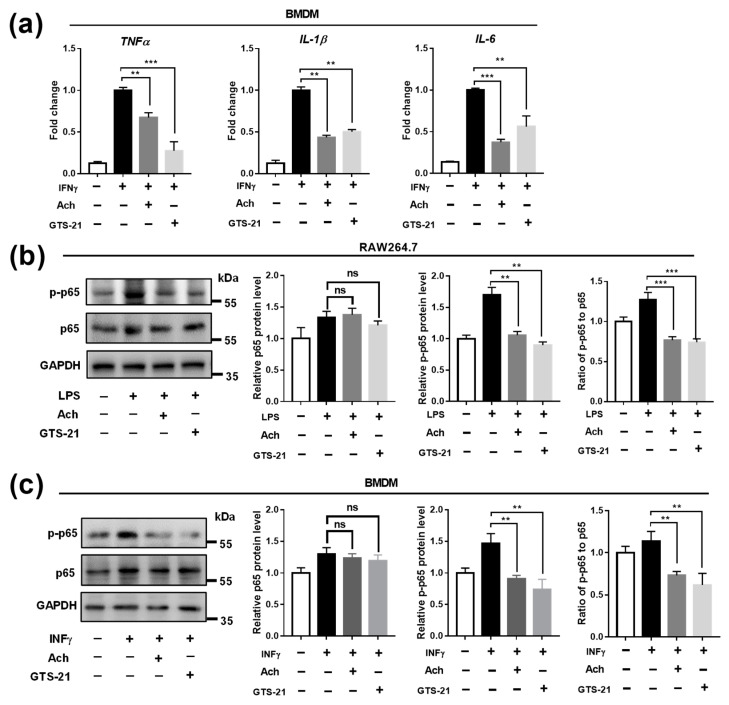

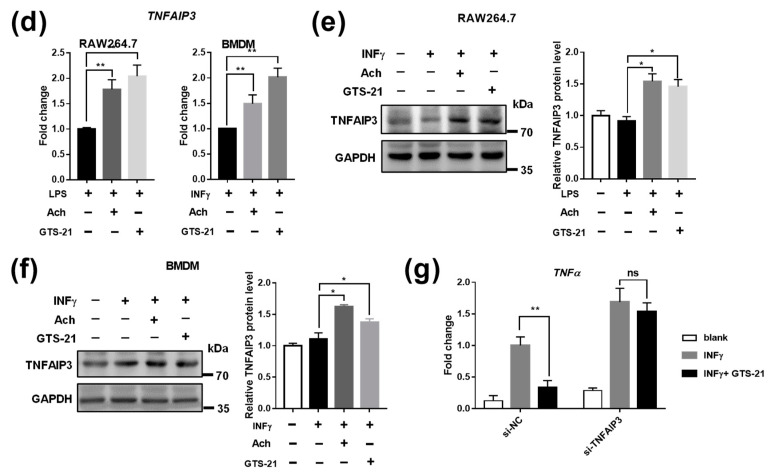
Effects of CAP agonists on M1 macrophage polarization. (**a**) The mRNA expression of M1 type marker genes (i.e., *TNFα*, *IL-1β*, and *IL-6*) were suppressed by the treatment of Ach and GTS-21 in INFγ induced BMDM. Data are presented as means ± SD (*n* = 3 biological replicates). ** *p* < 0.01, *** *p* < 0.001 as compared to INFγ induced cells. (**b**) Representative immunoblot images and quantitative analysis of inflammatory-related genes in LPS induced RAW264.7 cell treated with Ach and GTS-21. Data are presented as means ± SD (*n* = 3 biological replicates). ** *p* < 0.01 as compared to LPS-induced cells, ns indicates not significant. (**c**) Representative immunoblot images and quantitative analysis of inflammatory-related genes in INFγ induced BMDM treated with Ach and GTS-21. Data are presented as means ± SD (*n* = 3 biological replicates). * *p* < 0.05, ** *p* < 0.01 as compared to INFγ induced cells, ns indicates not significant. (**d**) The mRNA expression of *TNFAIP3* was upregulated by the treatment of Ach and GTS-21 in LPS/INFγ induced RAW264.7 and BMDM. Data are presented as means ± SD. ** *p* < 0.01 as compared to LPS/INFγ induced cells. (**e**,**f**) Representative immunoblot images and quantitative analysis of TNFAIP3 in RAW264.7 (**e**) and BMDM (**f**) treated with Ach and GTS-21. Data are presented as means ± SD (*n* = 3 biological replicates). * *p* < 0.05 as compared to INFγ induced cells. (**g**) GTS-21 inhibited expression of TNFα was restored by siRNA interference of TNFAIP3. Data are presented as means ± SD (*n* = 3 biological replicates). ** *p* < 0.01, ns indicates not significant.

The above finding prompted us to investigate whether CAP activity regulates the polarization of macrophages. To induce to M1 macrophage, the RAW264.7 cells were stimulated by LPS [57]. With the treatment of two CAP agonists, Ach and GTS-21, we found that the LPS-activated upregulation of pro-inflammatory genes such as *TNFα*, *IL-1β*, and *IL-6*, were dose-dependently suppressed (Supplementary Figure S2), suggesting an inhibitory effect of CAP agonist on M1 polarization. Based on these data, the concentrations of Ach at 100 μM and GTS-21 at 20 ng/mL were used in the subsequent experiment unless otherwise stated. Subsequently, the primary mouse BMDM cells were isolated from *Apoe^-/-^* mice and differentiated by M-CSF and INFγ for M1 macrophage [51]. Similarly, the induced M1 pro-inflammatory genes in BMDM were also significantly suppressed by both Ach and GTS-21 treatment (Figure 3a).

NFκB is known as a key transcriptional regulator that controls inflammatory signaling in M1 macrophage while blocking NFκB activity is efficient to suppress M1 activation [58,59]. Thus, we examined whether NFκB signaling was involved in the CAP pathway. The results showed that the expression of p-p65 but not total p65 was significantly inhibited by CAP agonists in M1-polarized RAW264.7 and BMDM cells (Figure 3b,c). Interestingly, we further found that Ach and GTS-21 treatment increased the expression of the tumor necrosis factor α induced protein 3 (TNFAIP3) (Figure 3d–f), a known negative feedback regulatory protein of NF-κB activity [60,61,62]. To validate whether TNFAIP3 mediated the function of GTS-21, siRNAs of TNFAIP3 were transfected into BMDM cells to knock down TNFAIP3 level (Supplementary Figure S3a). It was found that the inhibitive effects of GTS-21 on TNFα and IL-1β were markedly hindered by siTNFAIP3 (Figure 3g and Supplementary Figure S3b). Together, these results suggest that the inhibition of M1-like macrophage polarization by CAP agonists might be modulated by TNFAIP3 in suppressing the activation of p-p65.

### 3.4. Effects of CAP Agonists on M2 Macrophage Polarization

**Figure 4:**
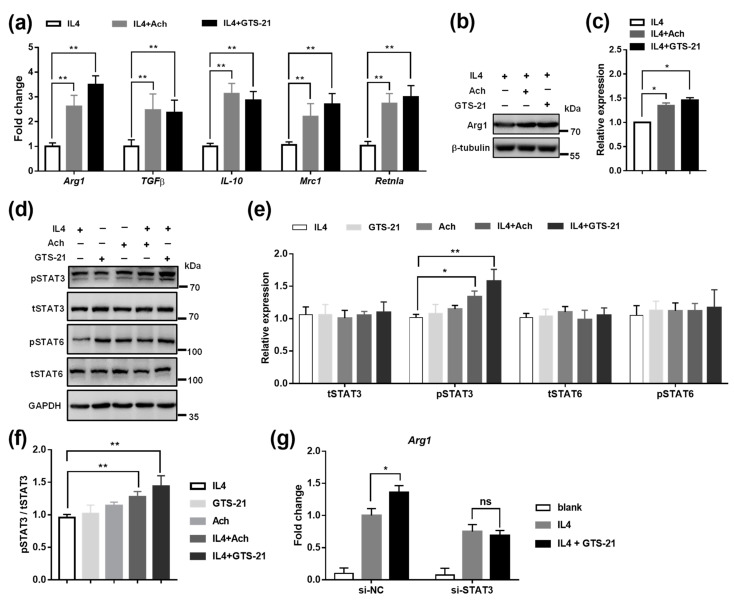
Effects of CAP agonists on M2 macrophage polarization. (**a**) The mRNA expression of M2 type marker genes (i.e., *Arg1*, *TGFβ*, *IL-10*, *Mrc1*, and *Retnla*) were upregulated by the treatment of Ach and GTS-21. Data are presented as means ± SD (*n* = 3 biological replicates). ** *p* < 0.01 as compared to IL4 induced cells. (**b**,**c**) Representative immunoblot images (**b**) and relative quantification (**c**) of Arg1 protein level in IL4-induced BMDM. Data are presented as means ± SD (*n* = 3 biological replicates). * *p* < 0.05 as compared to IL4 induced cells. (**d**,**e**) Representative immunoblot images (**d**) and relative quantification (**e**,**f**) of pSTAT3/tSTAT3, tSTAT3, pSTAT3, tSTAT6, and pSTAT6 in response to Ach and GTS-21 treatment in IL4 induced BMDM. Data are presented as means ± SD (*n* = 3 biological replicates). * *p* < 0.05, ** *p* < 0.01 as compared to blank cells. (**g**) siRNA interference of STAT3 inhibited GTS-21-induced expression of Arg1. Data are presented as means ± SD (*n* = 3 biological replicates). * *p* < 0.05, ns indicates not significant.

Next, we determined the role of CAP activity in M2 macrophage polarization. By the treatment of Ach or GTS-21, the expression of M2 macrophage marker genes, such as *Arg1*, *TGFβ*, *IL-10*, *Mrc1*, and *Retnla*, were significantly upregulated in RAW264.7 cells (Supplementary Figure S4). In addition, BMDM cells were also stimulated with IL4 for M2 macrophage activation, and we found that the CAP agonists enhanced the expression of M2 maker genes (Figure 4a–c), further confirming the positive role of CAP in promoting M2 macrophage polarization.

Since the STAT3 and STAT6 pathways are known to regulate M2 macrophage polarization [63,64,65,66], we then investigated whether these signalings are involved in the CAP pathway. The data revealed that both Ach and GTS-21 stimulation promoted the activation of pSTAT3 but not pSTAT6 in IL4-induced BMDM (Figure 4d–f). To confirm the involvement of STAT3 in mediating the function of GTS-21, we knocked down the STAT3 levels by transfecting siRNAs of STAT3 (Supplementary Figure S5a) and found that GTS-21 induced M2 marker gene Arg1 was blocked by siSTAT3 (Figure 4g). Thus, these findings indicate that CAP activation is likely to facilitate M2 macrophage polarization through the activation of STAT3 signaling.

### 3.5. Effects of CAP Activity on Macrophage Cholesterol Metabolism

**Figure 5:**
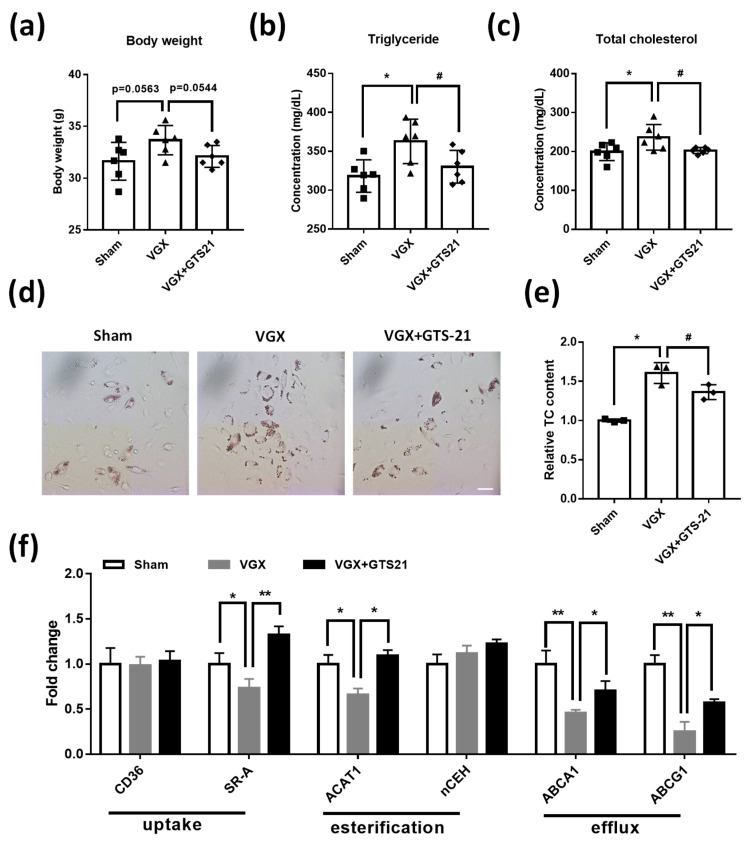
Effects of CAP activity on macrophage cholesterol metabolism in vivo. (**a**–**c**) The mice body weight (**a**), serum cholesterol (**b**), and triglyceride (**c**) were measured in the three groups of experimental mice, i.e., sham, VGX, and VGX + GTS-21. Data are presented as means ± SD (*n* = 6 mice). * *p* < 0.05 as compared to sham; # *p* < 0.05 as compared to VGX; ns indicates not significant. (**d**) The thioglycollate-elicited peritoneal macrophages were isolated from sham, VGX, and VGX + GTS-21 mice and then subjected to Oil-red O staining. Scale bar represent 50 μm (*n* = 20 image field). (**e**) Quantitative analysis cholesterol content in peritoneal macrophages isolated from the three experimental mice. Data are presented as means ± SD (*n* = 3 biological replicates). * *p* < 0.05 as compared to sham; # *p* < 0.05 as compared to VGX. (**f**) The mRNA expression analysis of genes associated with cholesterol metabolism in peritoneal macrophages isolated from the three experimental mice. Data are presented as means ± SD (*n* = 3 biological replicates). * *p* < 0.05, ** *p* < 0.01 as compared to macrophage isolated from VGX mice.

Macrophage-derived foam cells are the key immune cell for the cholesterol scavenging in the progress of AS [25,41], we then asked whether the CAP activity play a role in macrophage foam-cell cholesterol metabolism. No significant change in body weight was found in the three groups of experimental mice (Figure 5a). However, compared with the sham, VGX mice showed increased plasma cholesterol and triglyceride content, while in turn were reduced in VGX mice by the treatment of GTS-21 (Figure 5b,c). Next, we conducted an in vivo model for foam cell formation using thioglycollate-elicited peritoneal macrophages in the three experimental mice. The ORO staining revealed that extensive lipid droplets were exhibited in macrophages isolated from VGX mice rather than that isolated from VGX mice treated with GTS-21 (Figure 5d). Moreover, a similar change in cholesterol content was found in the peritoneal macrophage isolated from these mice (Figure 5e). Using real-time qPCR analysis, we detected several key genes that related to cholesterol uptake (i.e., CD36 and SR-A), esterification (i.e., ACAT1 and nCEH), and efflux (ABCA1 and ABCG1) [38,41,42]. It was found that the decreased expression of SR-A, ACAT1, ABCA1, and ABCG1 in macrophage from VGS mice were markedly rescued in macrophage from VGS mice treated with GTS-21 (Figure 5f).

### 3.6. Effects of CAP Agonists on ox-LDL Induced Macrophage Foam Cell Formation

**Figure 6:**
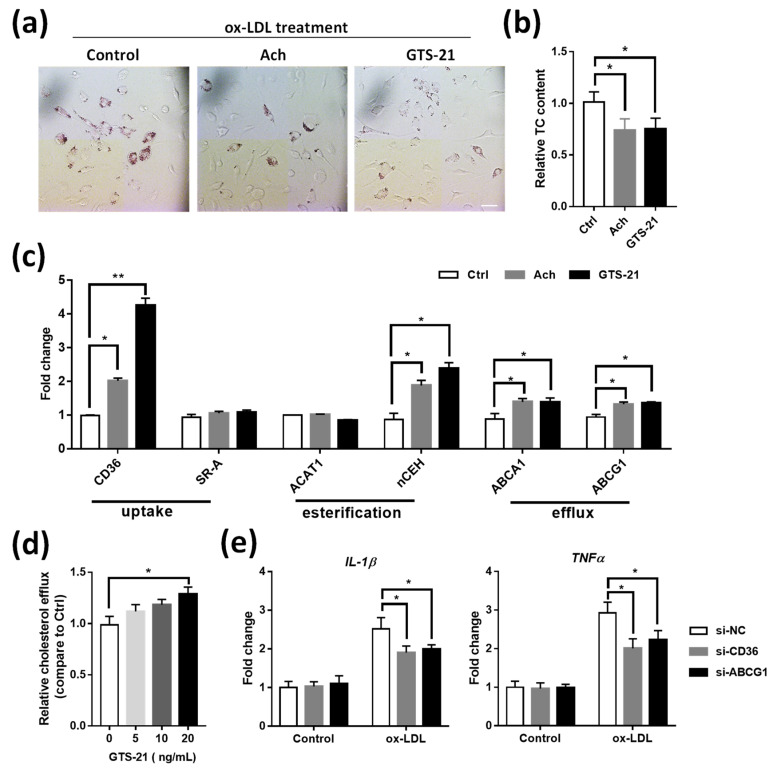
Effects of CAP agonists on ox-LDL induced macrophage foam cell formation. (**a**) Thioglycollate-elicited macrophages were treated with CAP agonist in the presence of ox-LDL to induce foam cell formation. The cells were then stained with ORO. Scale bar represents 50 μm. (**b**) Relative quantification analysis of total cholesterol in cultured peritoneal macrophages of (**a**). Data are presented as means ± SD (*n* = 3 biological replicates). (**c**) The mRNA expression analysis of genes associated with cholesterol metabolism in peritoneal macrophages treated with CAP agonist in the presence of ox-LDL. Data are presented as means ± SD (*n* = 3 biological replicates). * *p* < 0.05, ** *p* < 0.01 as compared to blank cells. (**d**) Relative cholesterol efflux mediated by different concentrations of GTS-21 treatment in peritoneal macrophages loaded with NBD-cholesterol. Data are presented as means ± SD (*n* = 3 biological replicates). (**e**) Effects of CD36 and ABCG1 on the inflammatory genes in peritoneal macrophages stimulated with ox-LDL. Data are presented as means ± SD (*n* = 3 biological replicates).

To further confirm the above finding, thioglycollate-elicited peritoneal macrophages were isolated from *Apoe^-/-^* mice to induce foam cell formation by ox-LDL stimulation. Compared with control, treatment of CAP agonists significantly decreased the lipid deposition in macrophage foam cells (Figure 6a,b). Further real-time qPCR analysis revealed that CAP agonists influence the expression of certain lipid metabolism-associated genes such as CD36, nCEH, ABCA1, and ABCG1 (Figure 6c). These data are consistent with the in vivo study, suggesting that the CAP plays an important role in the regulation of macrophage foam cell formation through genes related to cholesterol metabolism. Moreover, we found GTS-21 increased cholesterol efflux in macrophages dose-dependently (Figure 6d). Interestingly, the expression of inflammatory genes such as TNFα and IL-1β were significantly inhibited by the siRNA interference of CD36 or ABCG1 (Figure 6e and Supplementary Figure S5b,c), indicating that the inflammation effects were tightly associated with the lipid handling of macrophages.

## 4. Discussion

**Figure 7:**
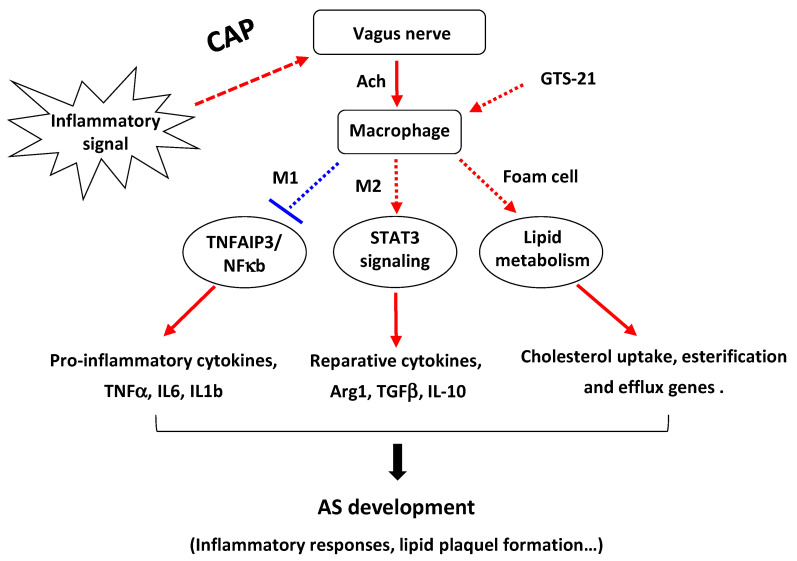
A schematic mechanism by which CAP regulates AS development. By receiving inflammatory signals, the vagus nerve can release neurotransmitter Ach to act on macrophages, leading to distinguished functions, i.e., the regulation of M1/M2 macrophage polarization and formation of foam cells, through coordinating their respective functional genes. CAP: cholinergic anti-inflammatory pathway; Ach: acetylcholine; AS: atherosclerosis.

Accumulative evidence points to a protective role of CAP activation in experimental models of inflammation and animal models of cardiovascular diseases, yet the underlying mechanisms still remain largely unknown [15,19,20]. In the present work, we provide a novel regulatory mechanism of CAP in AS pathogenesis, demonstrating that the activation of CAP can differentially regulate macrophage functions, i.e., the polarization of M1/M2 macrophages and formation of foam cells, through modulating their respective functional genes, thereby synchronously contributing to the development of AS pathogenesis (Figure 7).

A vast number of studies have well established that the activation of CAP, either by stimulation of the vagus nerve or by using the CAP agonist, can effectively attenuate inflammatory-related disease severity in animal models and clinical study [6,8,10]. For instance, electrical stimulation of the vagus nerve significantly reduces cytokine production and inhibits inflammatory responses and disease severity in experimental models of endotoxemia, sepsis, and colitis [1,67,68,69]. Moreover, vagus nerve stimulation has been used in patients with epilepsy and rheumatoid arthritis, leading to reduced TNF production and attenuated disease severity [10]. Similarly, the pharmacological application of CAP agonists (e.g., GTS-21 or AR-R17779) can also inhibit the release of cytokines and inflammatory conditions in AS animal models [11,15,20]. Consistent with these studies, our result in this work showed that blocking CAP activity by performing VGX resulted in increased proinflammatory cytokines production and atherosclerotic lipid plaque, indicating a detrimental role of VGX in AS progression. On the contrary, the treatment GTS-21 in VGX mice significantly rescued these deteriorative effects of VGX (Figure 1). Therefore, these findings imply that the vagus nerve is directly involved in the modulation of CAP activity to regulate AS pathogenesis, while the anti-atherosclerotic effects of GTS-21 could be due to its reactivation of CAP. In coordination with these findings, our in vitro study using RAW264.7 and primary BMDM cells further confirmed that GTS-21 treatment can substantially inhibit the expression of pro-inflammatory factors (Figure 3a and Supplementary Figure S2). Nevertheless, the exact role of GTS-21 in CAP and the related regulatory network should be further examined.

Although various immune cell types are involved, macrophages [70], which contribute to inflammation resolution, the formation, and regression of atherosclerotic plaques, play a central role in the pathophysiology of AS at every stage [25,26,28,29,71]. It has been shown that macrophage retention can be reversed in some models of atherosclerosis regression, leading to the identification of pathways that promote macrophage accumulation in, or egress from, the inflamed plaque [71,72]. These studies have shown that both the quantity and the phenotype of macrophages influence the inflammatory state of plaque. In this study, we demonstrated that alteration of CAP activity could essentially affect the number of M1 and M2 macrophages in atherosclerotic plaque and blood (Figure 2). Moreover, the in vitro study showed that treatment of Ach or GTS-21 suppressed the M1, but enhanced M2 macrophage polarization through the regulation of pro-inflammatory factors and reparative cytokines, respectively (Figure 3 and Figure 4). Interestingly, these results are tightly coordinated with the change of AS pathologic features (e.g., production of proinflammatory cytokines) in *Apoe^-/-^* mice responding to CAP activity (Figure 1). Thus, our findings indicate that stimulation of CAP could be a potential method to modulate M1 and M2 macrophage polarization for inflammation and plaque intervention in the development of AS. Moreover, we further displayed that TNFAIP3 and STAT3 might serve as key mediators acting downstream of CAP signaling to regulate M1/M2 macrophage functions (Figure 3d–g and Figure 4d–g), while the exact molecular mechanism by which CAP regulates the macrophage polarization should be further explored in future work.

As a major hallmark of atherosclerotic lesions, macrophage-derived foam cells play an important role in the occurrence and development of lipid plaque [41,42]. Excessive uptake of oxidized low-density lipoprotein (oxLDL), aberrant cholesterol esterification, and/or reduced cholesterol efflux lead to the deposition of esterified cholesterol ester in the cytoplasm of macrophages and subsequently the formation of foam cells [38,40,41]. In the present work, we showed that VGX increased, while VGX mice treated with GTS-21 decreased the number of foam cell formations in the peritoneal cavity (Figure 5). Moreover, in vitro study revealed that CAP agonists (i.e., Ach and GTS-21) also suppressed the accumulation of ox-LDL in peritoneal macrophages (Figure 6), thus these data suggest that CAP activity plays a role in regulating the formation of macrophage foam cells.

It is recognized that the balance of lipoprotein derived cholesterol is controlled by a series of lipid metabolism associated genes that regulate cholesterol influx (e.g., scavenger receptors such as CD36 and SR-A), esterification (e.g., ACAT1 and nCEH), and release (e.g., ABCA1 and ABCG1) in macrophage foam cells [38,39,40,41,42]. Our results illustrated that macrophages isolated from VGX mice showed decreased levels of SR-A, ACAT1, ABCA1, and ABCG1, while the expression of these genes was partially restored in macrophages isolated from VGX mice treated with GTS-21 (Figure 5). Consistently, we showed that CAP agonist treatment led to upregulation of CD36, nCEH, ABCA1, and ABCG1 in peritoneal macrophage (Figure 6). Consistently, the cholesterol efflux was also increased by GTS-21 treatment in peritoneal macrophages (Figure 6d). Thus, it is speculated that the increased expression of these genes by the activation of CAP may accelerate cholesterol metabolism in macrophages, which are coincident with the inhibitive effects of CAP on foam cell formation in this work. Nevertheless, more in-depth work should be taken to elucidate the exact underlying molecular mechanisms of how each step of lipid metabolism (i.e., cholesterol influx, esterification, and efflux) is regulated by GTS-21 and CAP activation.

Although the mechanisms of macrophage foam cell formation and inflammation have been extensively studied, it remains an intriguing question that whether the inflammation effects are caused by lipid handling of the macrophages. The previous study has indicated the primary response of macrophages to lipid stimulation is to trigger the inflammatory signaling, which then activates the lipid metabolism genes as a secondary response [73]. Moreover, the scavenger receptor, known to mediate the uptake of modified lipoprotein uptake, is also recognized to regulate various cellular functions and serve as pattern recognition receptors for stimuli that may contribute both to proinflammatory and anti-inflammatory forces in AS [74]. For instance, the deletion of SR-A1 or CD36 in *Apoe^-/-^* mice led to reduced signs of inflammation [75,76]. In this work, we found that pro-inflammatory genes (i.e., TNFα and IL-1β) were inhibited by the siRNA interference of CD36 or ABCG1 (Figure 6e), further confirming these cholesterol metabolism-related genes are involved in regulating macrophage inflammation. Nevertheless, whether the scavenger receptors also mediate the effects of CAP/GTS-21 on macrophage functions should be investigated in the future.

There are several limitations in the present study. Firstly, the application of GTS-21 was performed after the onset of interruption of CAP, i.e., either after VGX surgery in AS mice model or after the activation of macrophage by stimuli such LPS, IL4, and oxLDL. These data indicate a preventive effect of GTS-21 on AS development, while additional therapeutic studies should be conducted to investigate whether GTS-21 could recover or reverse the malformations in advanced, established atherosclerotic situations. Moreover, the exact role of GTS-21 involved in CAP activation and the related regulatory mechanism should be further examined in future works. Secondly, although the *Apoe^-/-^* mouse is currently the most popular and convenient animal model for AS research [77], this AS model will only partially reflect the human pathophysiologic features, of which the M1/M2 differentiation and foam cell development is a continuous, long-term procedure. Therefore, our finding that the activation of CAP by GTS-21 could attenuate the development of AS through regulating macrophage functions, need to be further verified in other animal models and clinical study. Last but not least, several additional experiments could be performed to give an insight into this work in the future. For instance, the IHC/IF staining could be used to quantify M1 and M2 macrophages, SMC content, and collagen content in plaque lesion; cholesterol efflux experiments could be conducted to validate the ability of GTS-21 in facilitating the efflux of lipids from macrophages.

## 5. Conclusions

In conclusion, our study provides direct evidence that CAP by vagus could never be essentially involved in the development of AS, namely the interruption of CAP by VGX aggravated, while reactivation of CAP ameliorated the inflammatory responses and pathological features in *AS* mice. Moreover, our results demonstrate that the beneficial effects of CAP on AS progression could be attributed to its regulation of macrophage M1/M2 polarization and foam cell formation. This study also suggests that activation of CAP through the vagus nerve or CAP agonists could be a potential strategy to serve as a therapeutic intervention for AS, although further validation in more animal models and clinical studies should be performed.

## Figures and Tables

**Table 1:** Primers sequences used in this study.

Gene Primer Name	Sequence (5′-3′)
*TNFα-F*	GCCTCTTCTCATTCCTGCTT
*TNFα-R*	TGGGAACTTCTCATCCCTTTG
*IL-1β-F*	TGGCAACTGTTCCTG
*IL-1β-R*	GGAAGCAGCCCTTCATCTTT
*IL-6-F*	CAAAGCCAGAGTCCTTCAGAG
*IL-6-R*	GTCCTTAGCCACTCCTTCTG
*Arg1-F*	AAGAATGGAAGAGTCAGTGTGG
*Arg1-R*	GGGAGTGTTGATGTCAGTGTG
*IL10-F*	AGGCGCTGTCATCGATTT
*IL10-R*	CACCTTGGTCTTGGAGCTTAT
*TGFβ-F*	CCTGAGTGGCTGTCTTTTGA
*TGFβ-R*	CGTGGAGTTTGTTATCTTTGCTG
*Mrc1-F*	CTCTGTTCAGCTATTGGACGC
*Mrc1-R*	CGGAATTTCTGGGATTCAGCTTC
*Retnla-F*	CTGGGTTCTCCACCTCTTCA
*Retnla-R*	TGCTGGGATGACTGCTACTG
*TNFAIP3-F*	ACAGGACTTTGCTACGACAC
*TNFAIP3-R*	CTGAGGATGTTGCTGAGGAC
*CD36-F*	GCGACATGATTAATGGCACAG
*CD36-R*	GATCCGAACACAGCGTAGATAG
*SRA-F*	GGGAACACTCACAGACACTG
*SRA-R*	CCCGATCACCTTTAACACCTG
*ACAT1-F*	AGCACACTGAACGATGGAG
*ACAT1-R*	CGCAAGTGGAAAATCAATGGG
*nCEH-F*	CTACGTGTACATCCCACTGC
*nCEH-R*	GATGAAATTCAGCGCGATCAG
*ABCA1-F*	TGACATGGTACATCGAAGCC
*ABCA1-R*	GATTTCTGACACTCCCTTCTGG
*ABCG1-F*	CCTTATCAATGGAATGCCCCG
*ABCG1-R*	CTGCCTTCATCCTTCTCCTG
*GAPDH-F*	AACAGCAACTCCCACTCTTC
*GAPDH-R*	CCTGTTGCTGTAGCCGTATT

## Data Availability

All data needed to evaluate the conclusions in the paper are present in the paper and/or the Supplementary Materials.

## Supplementary Materials

The following are available online at https://www.mdpi.com/article/10.3390/biomedicines9091150/s1, Figure S1: Validation of the development of atherosclerotic plaques in HFD-fed *Apoe*^-/-^ mice. The wild type (WT) and *Apoe*^-/-^ mice were fed with high fat food to induce the formation of atherosclerotic plaques in aorta. (a) Oil Red O staining of en face preparations of the thoracic aorta from WT and *Apoe*^-/-^ mice. Representative samples are shown. Scale bars represent 200 mm. (b) Quantitative analysis of the atherosclerotic plaque area in the aorta. Values represent mean ± SD (n = 3 mice). *** *p* < 0.001 as compared to WT. (c) The aortic arch from WT and *Apoe*^-/-^ mice were sectioned and stained with Oil Red O. Scale bar represent 100 μm. (d) Quantitative analysis of the atherosclerotic lipid lesion in aortic root (Average of 3–5 sections per mouse). n = 18–20 sections; Values represent mean ± SD. *** *p* < 0.001 as compared to WT. Figure S2: Ach and GTS-21 inhibit the expression of pro-inflammatory genes in LPS induced RAW264.7. The cells were treated with Ach or GTS-21 for 24 h in the presence of LPS, and then collected for the experiment. (a–c) The mRNA expression of TNFα (a), IL-1β (b) and IL-6 (c) were dose dependently depressed by the treatment of Ach. Data are presented as means ± SD (n = 3 biological replicates). * *p* < 0.05, ** *p* < 0.01, *** *p* < 0.001 as compared to LPS-induced cells. (d–f) The mRNA expression of TNFα (d), IL-1β (e) and IL-6 (f) were dose dependently depressed by the treatment of GTS-21. Data are presented as means ± SD (n = 3 biological replicates). * *p* <0.05, ** *p* < 0.01, *** *p* < 0.001 as compared to LPS-induced cells. Figure S3: (a) The mRNA expression of TNFAIP3 were determined in BMDM cells transfected with three siRNAs of TNFAIP3. (b) GTS-21 inhibited expression of IL-1 was prevented by siRNA interference of TNFAIP3 (3# siTNFAIP3). Data are presented as means ± SD (n = 3 biological replicates). * *p* < 0.05, ** *p* < 0.01, *** *p* < 0.001, ns indicates not significant. Figure S4: Ach and GTS-21 promoted the mRNA expression of M2 marker genes in IL4-induced RAW264.7. The cells were treated with Ach or GTS-21 in the presence of IL4 for 24 h, and then collected for qRTPCR analysis. (a–e) The mRNA expression of Arg1 (a), TGF (b), IL-10 (c), Mrc1 (d) and Retnla (e) were upregulated by the treatment of Ach (100 μm and GTS-21 (20 ng/mL). Data are presented as means ± SD (n = 3 biological replicates). * *p* < 0.05, ** *p* < 0.01 as compared to IL4 induced cells. Figure S5: The mRNA expression of STAT3 (a), CD36 (b) and ABCG1 (c) were determined in macrophages after the transfection of their respective siRNAs. Data are presented as means ± SD (n = 3 biological replicates). * *p* < 0.05, ** *p* < 0.01 as compared to si-NC.

## Abbreviations

CAP: (cholinergic anti-inflammatory pathway); AS, (atherosclerosis); VGX, (cervical vagotomy surgery); HFD, (high fat diet); BMDM, (bone marrow-derived macrophages); GTS-21, (Gainesville Tokushima scientists-21); Ach, (acetylcholine); IFN-γ, (interferon-γ); TNFAIP3, (tumor necrosis factor α induced protein 3); LPS, (lipopolysaccharide); ORO, (oil red O); ACAT1, (Acyl coenzyme A: cholesterol acyltransferase-1); nCEH, (neutral cholesteryl ester hydrolase); TGF (transforming growth factor)β.
