# Supplementary material for: The Cholinergic Anti-Inflammatory Pathway Attenuates the Development of Atherosclerosis in Apoe-/- Mice through Modulating Macrophage Functions
Source: Biomedicines. 2021 Sep 3;9(9):1150. doi: 10.3390/biomedicines9091150 (PMC8464862; doi:10.3390/biomedicines9091150)

Supplementary Figures

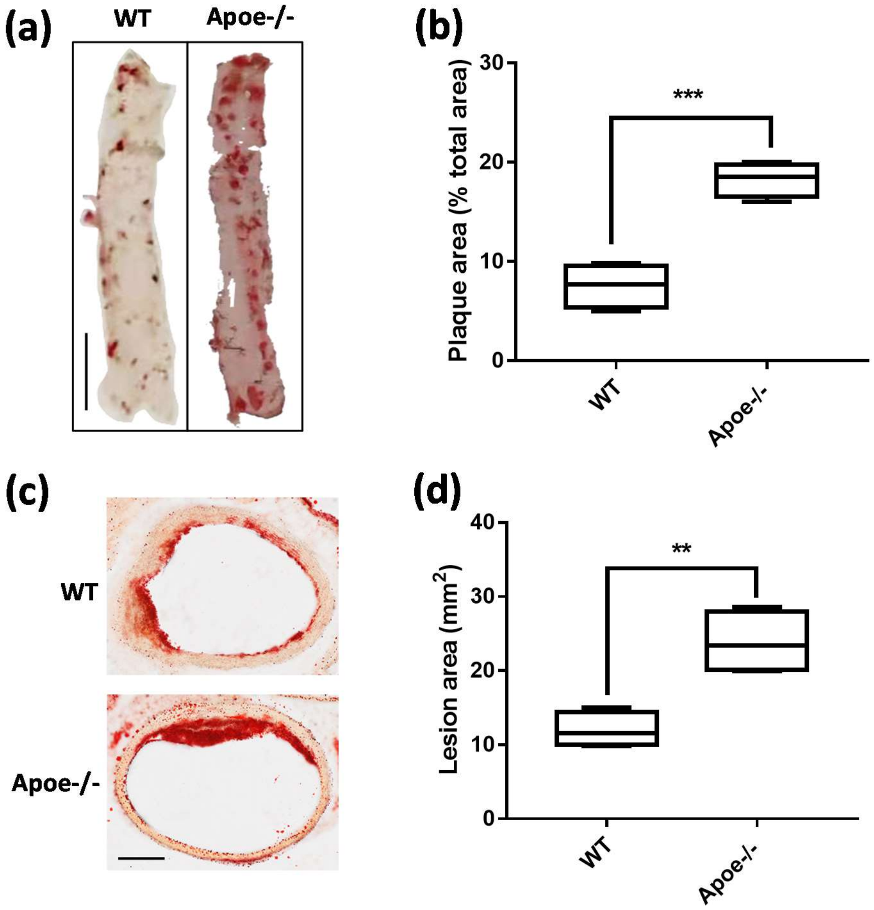

Supplementary Figure S1 Validation of the development of atherosclerotic plaques in HFD-fed *Apoe*$^{-/-}$ mice. The wild type (WT) and *Apoe*$^{-/-}$ mice were fed with high fat food to induce the formation of atherosclerotic plaques in aorta. (a) Oil Red O staining of en face preparations of the thoracic aorta from WT and *Apoe*$^{-/-}$ mice. Representative samples are shown. Scale bars represent 200 mm. (b) Quantitative analysis of the atherosclerotic plaque area in the aorta. Values represent mean ± SD (n = 3 mice). *** p <0.001 as compared to WT. (c) The aortic arch from WT and *Apoe*$^{-/-}$ mice were sectioned and stained with Oil Red O. Scale bar represent 100 µm. (d) Quantitative analysis of the atherosclerotic lipid lesion in aortic root (Average of 3–5 sections per mouse). n = 18–20 sections; Values represent mean ± SD. *** p <0.001 as compared to WT.

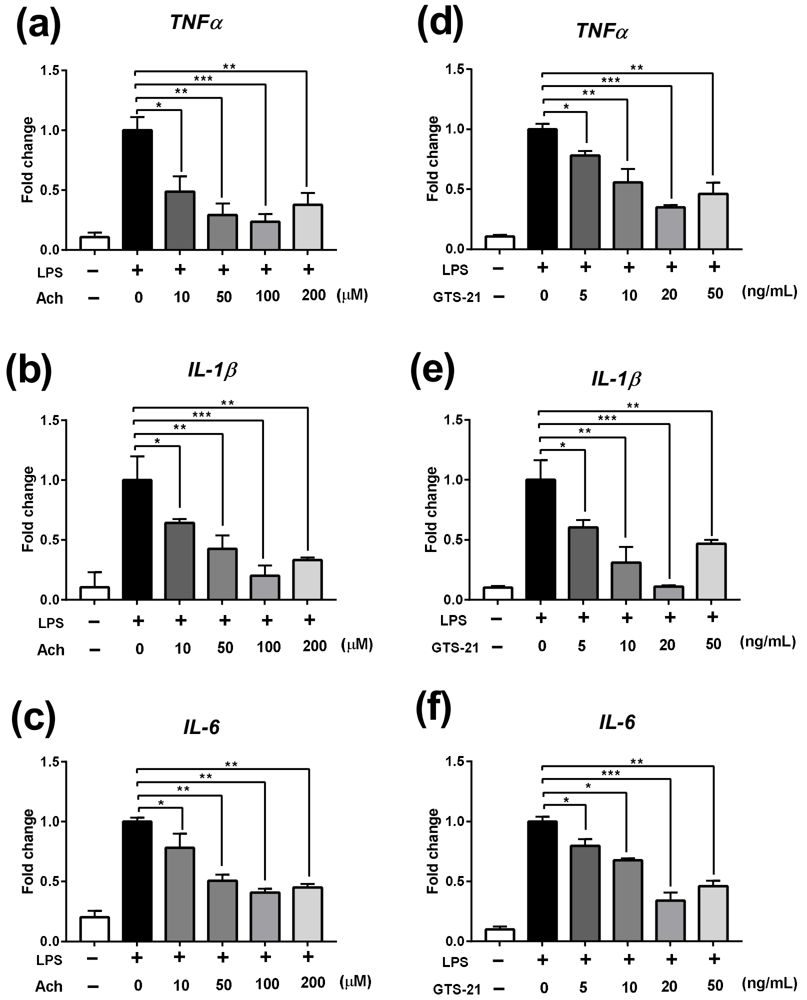

Supplementary Figure S2 Ach and GTS-21 inhibit the expression of pro-inflammatory genes in LPS induced RAW264.7. The cells were treated with Ach or GTS-21 for 24h in the presence of LPS , and then collected for the experiment. (a — c) The mRNA expression of TNF*α* (a), IL-1β (b) and IL-6 (c) were dose dependently depressed by the treatment of Ach. Data are presented as means ± SD (n = 3 biological replicates). * p <0.05, ** p <0.01, *** p <0.001 as compared to LPS-induced cells. (d — f) The mRNA expression of TNF*α* (d), IL-1β (e) and IL-6 (f) were dose dependently depressed by the treatment of GTS-21. Data are presented as means ± SD (n = 3 biological replicates). * p <0.05, ** p <0.01, *** p <0.001 as compared to LPS-induced cells.

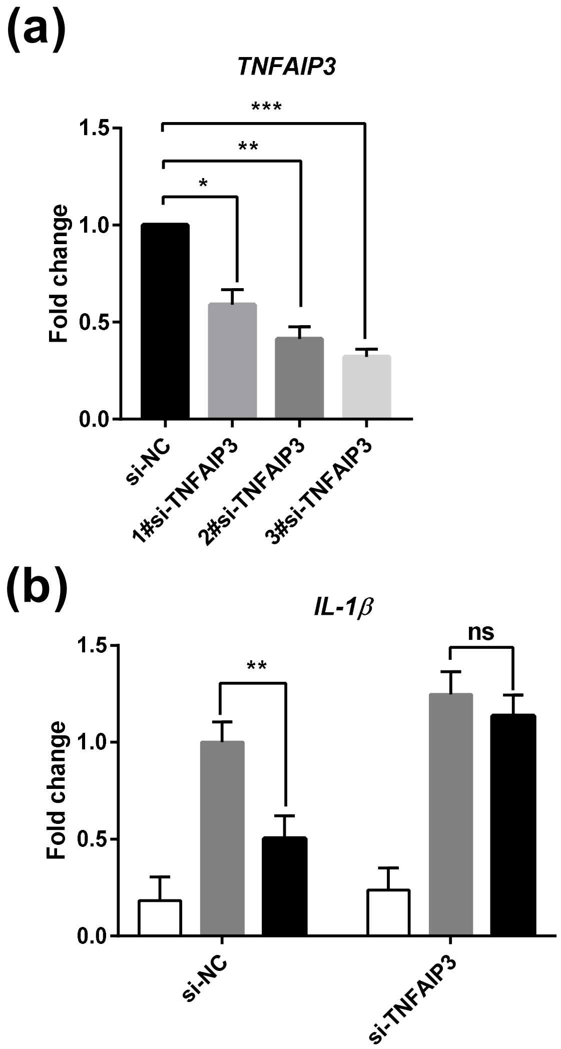

Supplementary Figure S3 (a) The mRNA expression of TNFAIP3 were determined in BMDM cells transfected with three siRNAs of TNFAIP3. (b) GTS-21 inhibited expression of IL-1β was prevented by siRNA interference of TNFAIP3 (3# siTNFAIP3). Data are presented as means ± SD (n = 3 biological replicates). * *p*<0.05, ** *p*<0.01, *** *p* <0.001, ns indicates not significant.

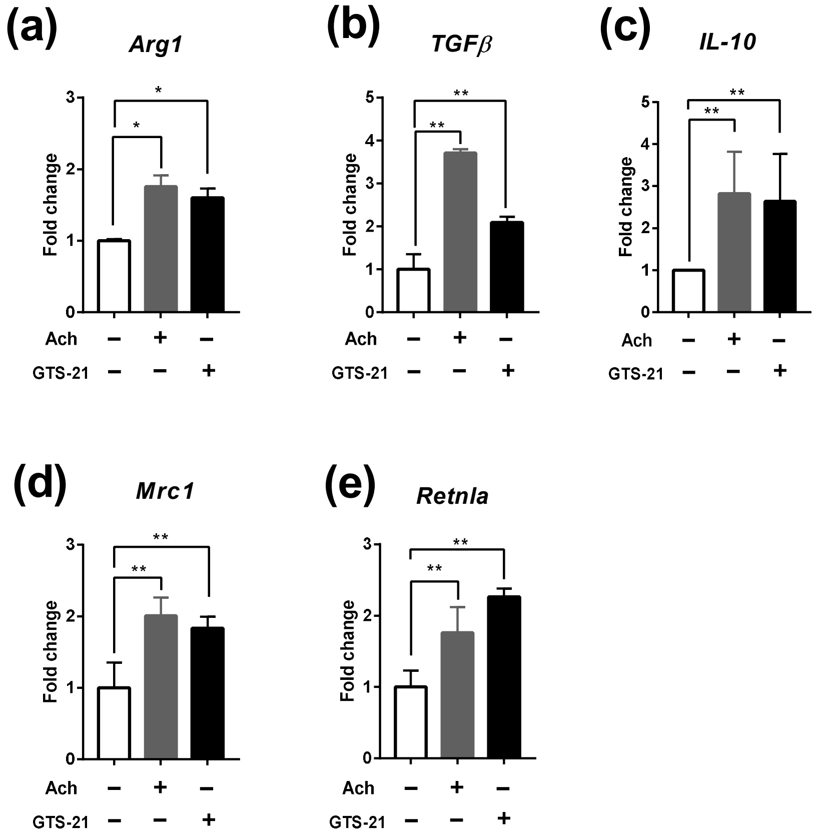

Supplementary Figure S4 Ach and GTS-21 promoted the mRNA expression of M2 marker genes in IL4-induced RAW264.7. The cells were treated with Ach or GTS-21 in the presence of IL4 for 24h, and then collected for qRTPCR analysis. (a — e) The mRNA expression of Arg1 (a), TGFβ (b), IL-10 (c), Mrc1 (d) and Retnla (e) were upregulated by the treatment of Ach (100 μm) and GTS-21 (20ng/mL). Data are presented as means ± SD (n = 3 biological replicates). * $p<0.05$, ** $p<0.01$ as compared to IL4 induced cells.

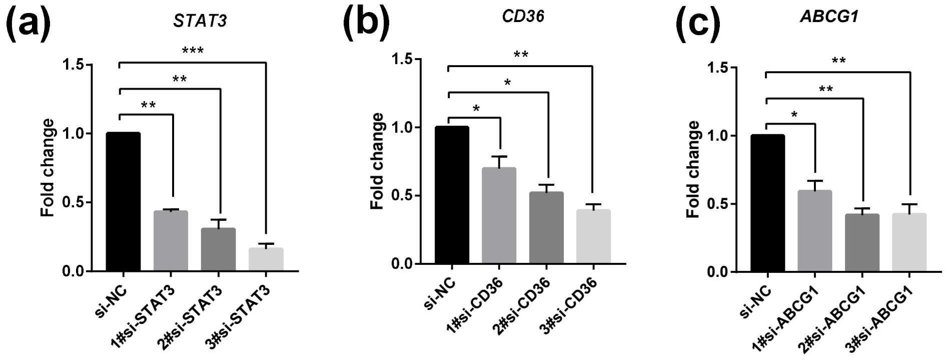

Supplementary Figure S5 The mRNA expression of STAT3 (a), CD36 (b) and ABCG1 (c) were determined in macrophages after the transfection of their respective siRNAs. Data are presented as means ± SD (n = 3 biological replicates). * p <0.05, ** p <0.01 as compared to si-NC.

Figures of replicates for experiment

A second western blot replicate for:

Figure 3b

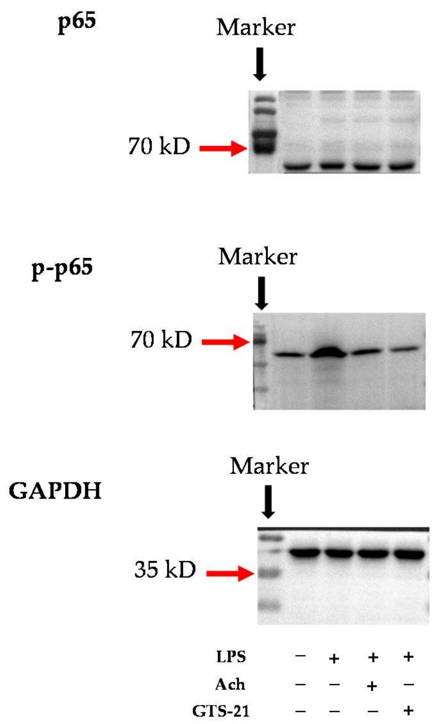

Figure 3c

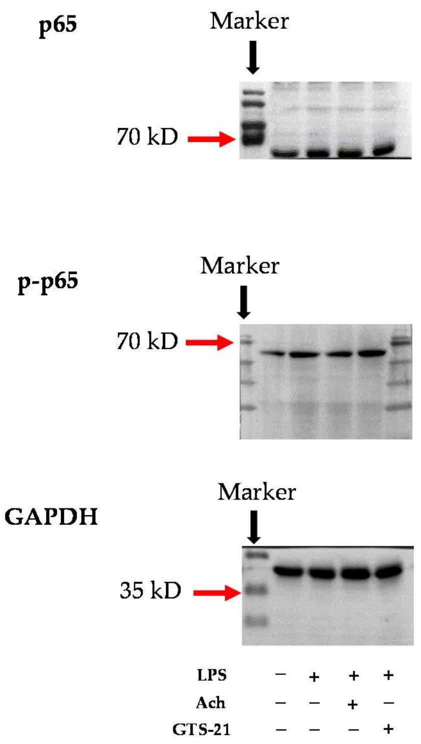

A second western blot replicate for:

Figure 3e

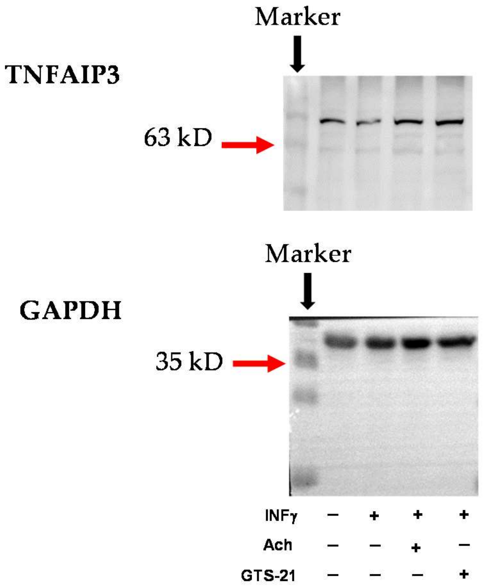

Figure 3f

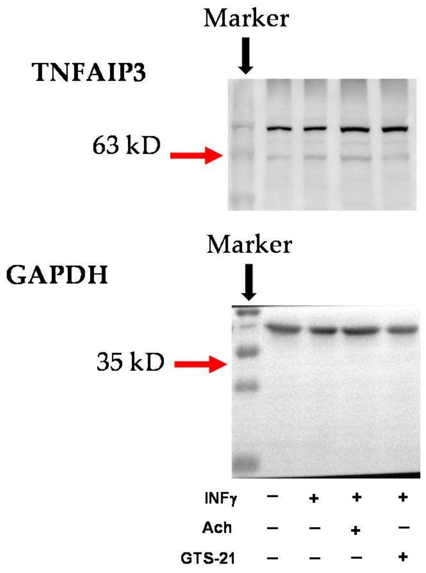

A second western blot replicate for:

Figure 4b

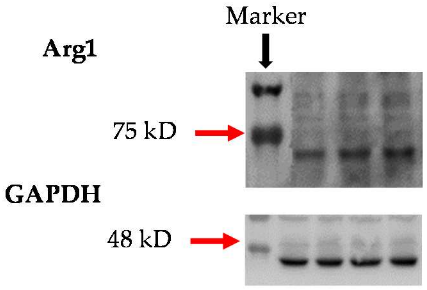

Figure 4d

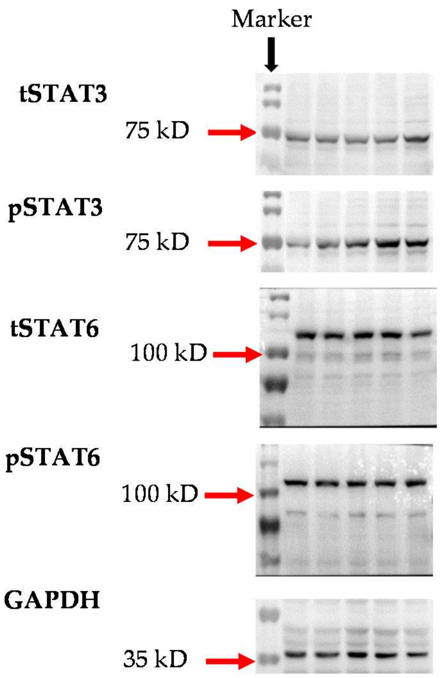

Supplement: Supplementary file 1 [file biomedicines-09-01150-s001.zip › biomedicines-1337486-supplementary.pdf]
